# Erratum to “Long-Term Treatment of Native LDL Induces Senescence of Cultured Human Endothelial Cells”

**DOI:** 10.1155/2017/4576985

**Published:** 2017-09-20

**Authors:** Sung-Tack Oh, Hoon Park, Hyun Joong Yoon, Sung Yeul Yang

**Affiliations:** ^1^Department of Obstetrics and Gynecology, Chonnam National University Medical School, 264 Seoyang-ro, Hwasun-eup, Jeollanam-do 58128, Republic of Korea; ^2^Department of Biochemistry, Chonnam National University Medical School, 264 Seoyang-ro, Hwasun-eup, Jeollanam-do 58128, Republic of Korea

In the article titled “Long-Term Treatment of Native LDL Induces Senescence of Cultured Human Endothelial Cells” [1], there were a number of language errors, due to publisher error. The corrected version of the article is shown below:

## Abstract

The study aimed to evaluate whether the treatment of primary cultured human endothelial cells with native low-density lipoprotein (nLDL) could induce their senescence and to uncover some of the putative mechanisms involved. For this purpose, human umbilical vein endothelial cells (HUVECs) were subcultured and/or continuously cultured with nLDL (0, 2, 5, and 10 *μ*g protein/mL), for up to 9 days. The results indicated that nLDL inhibited the proliferation of HUVECs by arresting the cell cycle at the G1 phase. The G1-arrested cells showed an increase in cytosolic senescence-associated-*β*-galactosidase (SA-*β*-Gal) activity, a biomarker of cellular senescence. The causative factor of the cellular senescence was nLDL itself and not oxidized LDL (oxLDL), since blocking the LDL receptor (LDLR) with the anti-LDLR antibody opposed the nLDL-induced increase of SA-*β*-Gal activity and the decrease of cellular proliferation. In addition, nLDL induced cellular senescence by inhibiting the phosphorylation of pRb (G1 arrest) via p53 as well as p16 signal transduction pathways. The G1 phase arrest of the senescent cells was not overcome by nLDL removal from the culture medium. Moreover, the nLDL-treated cells produced reactive oxygen species (ROS) dose- and time-dependently. These results suggested, for the first time, that long-term treatment with nLDL could induce the premature senescence of endothelial cells.

## 1. Introduction

Aging is one of the main risk factors for cardiovascular diseases. Aging of blood vessels is associated with the development of endothelial dysfunction [1, 2] and atherosclerosis [3–5]. According to recent studies, vascular endothelial cells' senescence, that is, vascular aging, might be a fundamental cause for the development of cardiovascular diseases [6–10]. However, the molecular mechanisms of vascular aging remain unclear.

Cellular senescence is a stress response phenomenon that results in (a) a permanent secession from the cell cycle and (b) the appearance of distinct morphological and functional changes associated with impaired cellular homeostasis [6]. Cellular senescence can be classified into two types according to the presence or the absence of telomere shortening [11, 12]. Cellular senescence resulting from repeated cellular replication is called “replicative senescence” [13, 14]. In this type of senescence, the progressive shortening of telomeres, by end-replication problems, and their eventual dysfunction are considered as fundamental mechanisms of senescence induction. Another type of cellular senescence results from acute senescence response to various stressful conditions, such as intracellular oxidative stress or persistent mitogenic stimulation. This second type of cellular senescence is known as “stress-induced premature senescence” and is not accompanied by telomere shortening [15–17]. Both types of cellular senescence can occur in living organisms and are associated with diseases and aging of individuals [18–20].

Oxidized low-density lipoprotein (oxLDL) can exert various unfavorable effects on vascular endothelial cells, such as the impairment of endothelial nitric oxide formation [21], induction of endothelial expression of adhesion molecules [22], induction of superoxide anion formation from the vascular tissue [23], and induction of apoptosis [24] and senescence [25] of endothelial cells. Although oxLDL plays an important role in cellular senescence, the oxLDL levels in the blood of healthy adults, measured by an ELISA assay with a specific antibody, are about 0.12~0.13 ng/*μ*g LDL apolipoprotein B, which is <0.02% of total LDL [26, 27].

Actually, native LDL (nLDL), an LDL particle free of any modification induced by any agent within the blood, is hardly oxidized due to active circulating antioxidants; in addition, Kupffer cells and sinusoidal endothelial cells in the liver rapidly remove the oxLDL already produced [28, 29]. Circulating nLDL is most probably oxidized within the vascular wall and diffuses into the blood [30, 31]. These data are suggestive of a negligible contribution of oxLDL to the in vivo development of atherosclerosis and cellular senescence in the absence of pathological conditions, such as hyperlipidemia or diabetes mellitus.

Native LDL could potentially promote the recruitment of monocytes [32] and expression of cell adhesion molecules [33, 34] by disturbing the lipid dynamics of the endothelial cell membrane. These effects of nLDL on vascular endothelial cells could lead to the development of atherosclerosis [35]. However, it is unclear whether nLDL could contribute to the development of cellular senescence.

Native LDL-induced endothelial dysfunction and expression of cell adhesion molecules in endothelial cells match the phenotypes of vascular aging, that is, endothelial cell senescence [18, 36]. Hence, most likely, nLDL could induce the senescence of endothelial cells. Thus, we determined whether nLDL could induce the senescence of primary cultured human endothelial cells (HUVECs).

## 2. Materials and Methods

### 2.1. Materials

HUVECs (catalog number MC1133) were purchased from Modern Cell & Tissue Technology (Seoul, Korea) and microvascular endothelial cell medium-2 (EGM-2) was purchased from Cambrex (East Rutherford, NJ, USA). RIPA lysis buffer and protease inhibitor cocktail were purchased from Santa Cruz Biotechnology (Santa Cruz, CA, USA). West-ZOL® (plus) was purchased from iNtRON Biotechnology (Seongnam, Korea). The EZ-Cytox® cell viability assay kit was purchased from Daeil Lab Service (Seoul, Korea). X-Gal (5-bromo-4-chloro-3-indolyl-*β*-D-galactoside) was purchased from Amersham (Buckinghamshire, UK). Propidium iodide (PI) was purchased from Invitrogen (Carlsbad, CA, USA). *β*-Mercaptoethanol was purchased from Merck (Darmstadt, Germany). RNase A was purchased from Qiagen (Hilden, Germany). LDL, trypsin/EDTA solution, 4-methylumbelliferyl-*β*-D-galactopyranoside (MUG), 3-{(3-cholamidopropyl)dimethylammonio}-1-propanesulfonate hydrate (CHAPS), phenylmethanesulfonyl fluoride (PMSF), PVDF membrane, and all other reagents were purchased from Sigma (St. Louis, MO, USA).

Various antibodies were used in this study. Anti-human pRb and anti-human phospho-pRb (pS807/pS811) monoclonal antibodies were purchased from BD Biosciences (San Jose, CA, USA). Polyclonal antibodies against p16-INK4A, cyclin E2, and p53 and monoclonal antibodies against cyclin D1 and p21-CIP1 were purchased from Cell Signaling Technology (Danvers, MA, USA). Monoclonal antibodies against LDL receptor (LDLR), CDK2 and, CDK4 were purchased from Santa Cruz Biotechnology (Santa Cruz, CA, USA). Anti-actin polyclonal antibody and HRP-conjugated anti-rabbit or anti-mouse secondary antibodies were purchased from Sigma (St. Louis, MO, USA).

### 2.2. Cell Culture

HUVECs were cultured in EGM-2 medium at 37°C under a humidified atmosphere and 5% CO_2_. The cells were either subcultured or cultured continuously on a Petri dish for up to 9 days. Young HUVECs [population doubling level (PDL), 12~15] were inoculated on a Petri dish, stabilized by overnight incubation, and then treated with various concentrations of nLDL (0, 2, 5, or 10 *μ*g protein/mL). Media exchange and nLDL treatment were performed concomitantly at intervals of 3 days. For each subculture, the same number of cells were reinoculated on the same-sized Petri dish. After 3 days of nLDL treatment, the cells were washed thrice with PBS and treated with 0.25% trypsin/EDTA solution for 3 min. The detached cells were harvested, washed thrice with PBS, and used for various assays.

We cultured the cells by the subculture system by default. Both culture systems used here have disadvantages. Although the subculture system is commonly used, it is stressful to the cells to be harvested and inoculated every 3 days. The continuous culture system causes another type of stress to the cells as a consequence of the inoculation with a lesser number of cells. To eliminate the variables that could occur from each culture system, we used both a subculture system and a continuous culture system for some critical assays.

### 2.3. Determination of Cellular Proliferation

The cellular proliferation of HUVECs was analyzed by using a WST-1 (water-soluble tetrazolium salt-1) based cell viability assay kit (EZ-Cytox) [37]. The nLDL-treated cells (1.0 mL) were harvested with trypsin/EDTA, mixed with 100 *μ*L/mL of EZ-Cytox solution, and incubated at 37°C for 2 hr. The mixture was shaken for 1 min and 200 *μ*L of the mixture was transferred to a 96-well microplate. Absorbance of these samples was read at 450 nm with a microplate reader (Epoch, Winooski, VT, USA).

This assay was carried out in the cells either cultured continuously or subcultured. For subculture, the cells on a Petri dish (6  ×  10^4^ cells; *ϕ*, 35 mm dish) were subcultured twice concomitantly with nLDL treatment. Cellular proliferation was assayed at the last day of each subculture and expressed as the relative changes in absorbance of the nLDL-treated cells against that of the nLDL-untreated cells of the same culture duration. For continuous culture, the cells on a Petri dish (5 × 10^3^ cells; *ϕ*, 35 mm dish) were cultured on the same dish for up to 9 days with nLDL treatment and media exchange. Cellular proliferation was assayed every day and expressed as the relative changes in absorbance of each group of cells to that of the first day inoculated cells without nLDL treatment.

### 2.4. Determination of Cellular Senescence

#### 2.4.1. SA-*β*-Gal Activity Assay

The senescence of HUVECs was evaluated by the quantitative SA-*β*-Gal assay using cell extracts [38]. SA-*β*-Gal activity was measured by quantifying the generation rate of 4-methylumbelliferone (4-MU), the fluorescent hydrolysis product of MUG. Young HUVECs (PDL, 12~15) on a Petri dish (1.0 × 10^5^ cells; *ϕ*, 10 cm dish) were subcultured and treated with various concentrations of nLDL (0, 2, 5, or 10 *μ*g protein/mL) for up to 9 days. On the last day of each subculture, the cells were washed 6 times with PBS to remove residual growth medium protein that could interfere with subsequent cellular protein determination. The cells were then lysed in 450 *μ*L of 1 × lysis buffer [5 mM CHAPS, 40 mM citric acid, 40 mM sodium phosphate, 0.5 mM benzamidine, and 0.25 mM PMSF (pH 6.0)]. The lysate was scraped, transferred to a microtube, vortexed vigorously, and centrifuged at 12,000 ×g for 5 min at 4°C. The supernatant was kept on ice until further use. Reaction buffer at 2 × strength [40 mM citric acid, 40 mM sodium phosphate, 300 mM NaCl, 10 mM *β*-mercaptoethanol, and 4 mM MgCl_2_ (pH 6.0)] was mixed with 1.7 mM of MUG (SA-*β*-Gal substrate) immediately prior to its use. The reaction buffer containing MUG (45 *μ*L) was mixed with clarified lysate (30 *μ*L) and 1 × lysis buffer (15 *μ*L). Incubation lasted 1 hr, at 37°C in a water bath. After the incubation period, the reaction mixture was added to 400 mM sodium carbonate stop solution (900 *μ*L) and stored at 4°C until measurement of fluorescence. An aliquot of the carbonate-stopped reaction mixture was transferred to a 96-well plate (150 *μ*L/well) and fluorescence was read with a fluorescence microplate reader (VersaMax, Molecular Devices Corp., Philadelphia, PA, USA) with excitation at 360 nm and emission at 465 nm. The SA-*β*-Gal activity was calculated based on the fluorescence intensity of 4-MU/*μ*g protein and expressed as the relative activity of the nLDL-treated group to the untreated group of the same subculture duration.

#### 2.4.2. SA-*β*-Gal Staining

The cellular senescence was also evaluated by the SA-*β*-Gal activity-staining assay [39]. Using the same subculture conditions described in Section 2.4.1, and when appropriate, the cells were fixed with 3% (v/v) formaldehyde in PBS for 3~5 min and washed thrice with PBS. The washed cells were stained with SA-*β*-Gal staining solution [1 mg/mL X-Gal, 40 mM citric acid in 40 mM sodium phosphate buffer (pH 6.0), 5 mM potassium ferricyanide, 5 mM potassium ferrocyanide, 150 mM NaCl, and 2 mM MgCl_2_] at 37°C for 16 hr under light protection. The stained cells were washed twice with PBS and coated with 70% glycerol in PBS. The cells were then observed under a phase-contrast microscope (TS100F, Nikon, Tokyo, Japan).

### 2.5. Cell Cycle Analysis

The cell cycle of the nLDL-treated HUVECs was analyzed according to the flow cytofluorometric assay using PI staining [40]. Briefly, the cells on a Petri dish (1.0 × 10^5^ cells; *ϕ*, 10 cm dish) were subcultured and treated with nLDL (0, 2, 5, or 10 *μ*g protein/mL) for up to 9 days. When appropriate, the cells were harvested with trypsin/EDTA, washed thrice with PBS, and suspended in 300 *μ*L of PBS. The washed cells were fixed in 70% ice-cold ethanol for 1~2 hr and washed thrice by centrifuging at 700 ×g for 5 min in cold PBS. The fixed cells were treated with 50~100 *μ*L of RNase A (100 *μ*g/mL) for 15 min and then by 100 *μ*L of PI (50 *μ*g/mL) for 15 min under light protection. Cell cycle was analyzed with a FACSCalibur® flow cytometer (BD Biosciences, San Jose, CA, USA) with excitation at 535 nm and emission at 617 nm.

### 2.6. Western Blot Analysis

#### 2.6.1. Blocking of LDLR with Antibody

To block LDLR, the cells on a Petri dish (2 × 10^4^ cells; *ϕ*, 35 mm dish) were pretreated with anti-LDLR antibody (20 *μ*g protein/mL) for 1 hr at 4°C. Subsequently, the cells were treated with nLDL (10 *μ*g protein/mL) and cultured for up to 6 days. Senescence induction was analyzed by SA-*β*-Gal activity assay in subcultured cells and cellular proliferation by tetrazolium salt staining in continuously cultured cells every 3 days.

#### 2.6.2. Analysis of Cell Cycle-Regulating Proteins

Cell cycle-regulating proteins in HUVECs were analyzed by Western blot. As described in Section 2.6.1, the cells were subcultured for up to 6 days and lysed in the RIPA lysis buffer (50 mM Tris-HCl, 150 mM NaCl, 1% NP-40, 0.5% sodium deoxycholate, 0.1% SDS, and 0.004% sodium azide) containing 1 mM PMSF, 0.5 mM sodium orthovanadate, and a protease inhibitor cocktail (10~20 *μ*L per 1 mL lysis buffer). The mixtures were incubated at 4°C for 30 min and centrifuged at 12,000 ×g for 15 min at 4°C. The supernatants were stored at −80°C until further use.

Cell lysates (40 *μ*g protein) were separated by 8~15% gradient polyacrylamide gel electrophoresis and transferred to a PVDF membrane. The membrane was blocked with 5% skimmed milk in TBS-T buffer (Tris buffered saline-Tween-20 buffer; 137 mM NaCl, 2.7 mM KCl, and 0.1% Tween-20 in 25 mM Tris-base buffer, pH 7.4) at room temperature for 1 hr and incubated overnight with primary antibody at 4°C. After washing thrice in TBS-T buffer, the blot was incubated with the secondary antibody [HRP-conjugated anti-rabbit (1 : 2,000) or anti-mouse (1 : 5,000)] for 2 hr at room temperature. The antibody-specific proteins were detected using the West-ZOL (plus) Western blot detection system (iNtRON Biotechnology, Seongnam, Korea). The primary antibodies were used in the following dilutions: anti-p53 (1 : 1,000), anti-p21-CIP1 (1 : 1,000), anti-p16-INK4A (1 : 1,000), anti-CDK2 (1 : 1,000), anti-CDK4 (1 : 1,000), anti-cyclin E2 (1 : 1,000), anti-cyclin D1 (1 : 1,000), anti-pRb (1 : 250), anti-human pS807/pS811-pRb (1 : 250), and anti-actin (1 : 2,500).

### 2.7. Measurement of Intracellular ROS

ROS generation in the nLDL-treated HUVECs was analyzed using the method of Royall and Ischiropoulos [41] to evaluate whether intracellular ROS were implicated in the premature senescence of the cells. Young HUVECs on a culture dish (1.0 × 10^5^ cells; *ϕ*, 10 cm dish) were subcultured and treated with various concentrations of nLDL (0, 2, 5, or 10 *μ*g protein/mL) for up to 9 days. When appropriate, the cells were washed twice with HBSS and incubated at 37°C for 1 hr in a reaction mixture containing 11 *μ*M DCF-DA and 10 mM Hepes, pH 7.4, in phenol-red free EGM-2 medium. After incubation, the cells were washed twice with HBSS, supplemented with 1.5 mL of sonication buffer [50 mM potassium phosphate buffer (pH 7.0), 0.1 mM EDTA, and 0.1% CHAPS], and harvested by scraping. The cells were lysed by sonication (frequency, 24 Hz; duration, 10 sec/cycle; repetition, 6 cycles; time interval, 10 sec). An aliquot of supernatants (200 *μ*L/well) was transferred to a 96-well plate and fluorescence was measured with a fluorescence microplate reader at excitation 502 nm and emission 523 nm. Intracellular content of ROS was expressed as the relative fluorescence intensity/*μ*g protein of the nLDL-treated group to an untreated group of the same subculture duration.

Intracellular generation of ROS was also observed under fluorescent microscopy. DCF-DA-treated HUVECs were washed with HBSS twice and observed with an inverted fluorescence microscope (TE2000-U, Nikon, Japan) at 100x magnification.

### 2.8. Protein Quantification and Statistical Analysis

The protein concentration of each sample was measured using a BCA® protein assay kit (Pierce Biotechnology, Rockford, IL, USA). Bovine serum albumin was used as a protein standard.

Statistical analysis was performed by using SPSS Statistics 19 (IBM Corp., Armonk, NY, USA). The dose- and time-dependent differences between the groups were analyzed by repeated measures ANOVA assay and simple differences between the data were analyzed by independent *t*-test. *p* < 0.05 was considered statistically significant. Data was expressed as mean ± standard deviation (SD).

## 3. Results

### 3.1. Native LDL Inhibited Proliferation of HUVECs

The cellular proliferation assay was carried out to evaluate the effect of low concentrations of nLDL (0, 2, 5, or 10 *μ*g protein/mL) on the proliferation of cultured HUVECs (Figure 1). The effect of nLDL treatment on cellular proliferation was analyzed in both a subculture system (Figure 1(a)) and a continuous culture system (Figure 1(b)). Native LDL treatment significantly inhibited cell proliferation dose- and time-dependently in both culture systems (*p* < 0.01). Cells treated with nLDL for 3 days in a subculture system showed no significant inhibition of cellular proliferation, except at 10 *μ*g protein/mL (*p* < 0.05). Nevertheless, cells treated with nLDL for 6 or 9 days showed a significant inhibition of cellular proliferation (*p* < 0.01) for all concentrations tested. The inhibition of cell proliferation occurred after 2 days of nLDL treatment in a continuous culture system. The trypan blue assay of the nLDL-treated cells showed that cell death in this study was negligible (data not shown). These results showed that treatment with low concentrations of nLDL could inhibit the proliferation of cultured HUVECs in a dose- and time-dependent way.

### 3.2. Native LDL-Induced Senescence of HUVECs

Next, we evaluated the role of senescence of HUVECs in the nLDL-induced inhibition of cellular proliferation. The cells were treated with low concentrations of nLDL (0, 2, 5, and 10 *μ*g protein/mL) and cellular senescence was analyzed by quantitative assay (Figure 2(a)), as well as staining (Figure 2(b)) of SA-*β*-Gal activity in a subculture system for up to 9 days. Native LDL significantly increased the enzyme activity dose- and time-dependently in the quantitative assay (*p* < 0.01). Native LDL also increased staining of the enzyme activity. The SA-*β*-Gal activity of each nLDL-treated group was also compared with the respective nLDL-untreated group by independent *t*-test. The SA-*β*-Gal activity was significantly increased at 3, 6, and 9 days of all concentrations of the nLDL treatment (*p* < 0.01). The increased SA-*β*-Gal activity preceded the decreased cellular proliferation for the lower nLDL concentrations. These results suggested that the nLDL-induced inhibition of cellular proliferation could, at least partly, result from cellular senescence.

### 3.3. Native LDL-Induced Senescent Cells Were Arrested at G1 Phase of Cell Cycle

In the next experiment, we analyzed the change in the distribution of cell cycle phase of the nLDL-induced senescent HUVECs, in a subculture system (0, 2, 5, and 10 *μ*g protein/mL) for up to 9 days (Figure 3). Cell cycle analysis with flow cytometry after PI staining indicated that the distribution of G1 phase cells was significantly increased (*p* < 0.01) and the distribution of S and G2/M phase cells was significantly decreased (data not shown, *p* < 0.01) in a dose- and time-dependent way. The distribution of G1 phase cells at each nLDL-treated group was also compared with the respective nLDL-untreated group by independent *t*-test. The distribution of G1 phase cells was significantly increased at all concentrations of nLDL tested (*p* < 0.01). These results indicated that the nLDL-induced senescent HUVECs were arrested at the G1 phase of cell cycle.

### 3.4. Native LDL-Induced Cellular Senescence Resulted from NLDL Itself

To confirm that the nLDL-induced cellular senescence in HUVECs did not result from oxLDL generated from nLDL during in vitro incubation, we pretreated the cells with the monoclonal antibody against LDLR (anti-LDLR antibody) to block cellular LDLR before nLDL treatment (10 *μ*g protein/mL). The SA-*β*-Gal activity assay (Figure 4(a)) was carried out in subcultured cells and the cellular proliferation assay (Figure 4(b)) was carried out in continuously cultured cells. Both assays were performed at the cells cultured for up to 6 days, since 10 *μ*g protein/mL nLDL showed nearly maximum effect at 6 days (Figures 1(a) and 2(a)). The effect of nLDL on both cellular senescence parameters was significantly and negatively modulated by anti-LDLR antibody pretreatment at independent *t*-test (*p* < 0.01) as well as repeated measures ANOVA assay (*p* < 0.01). These results suggested that the nLDL-induced cellular senescence of HUVECs resulted from nLDL itself, and not oxLDL.

### 3.5. Cellular Senescence by NLDL Was Induced via Both p53 and p16-pRb Signal Pathways

To evaluate the signal transduction pathway involved in senescence induction with nLDL (10 *μ*g protein/mL), we conducted Western blot analysis for some cell cycle-regulating proteins at the last day of each subculture (Figure 5) for up to 6 days. As expected, the content of p53, p21, and p16 proteins was significantly increased in the nLDL-induced senescent cells (Figure 5(b1)). Two cyclin/CDK complexes such as CDK4/6-cyclin D and CDK2-cyclin E are known to phosphorylate pRb to overcome G1 arrest. The protein content of these complexes, that is, CDK2, cyclin E2, CDK4, and cyclin D1, was significantly decreased in the senescent cells (Figures 5(b2) and (b3)); and, as a result, the phosphorylation of pRB was also significantly inhibited (Figure 5(b4)). These results suggested that the nLDL-induced cellular senescence in HUVECs could result from the inhibition of pRb phosphorylation (i.e., G1 arrest of cell cycle) by the inhibition of the two cyclin/CDK complexes (CDK4/6-cyclin D and CDK2-cyclin E) via both the p53 and p16 signal transduction pathways. Pretreatment with the anti-LDLR antibody restored in varying degrees the changes in the levels of the cell cycle-regulating proteins induced by nLDL treatment (Figure 5). These results corroborated the results of Figure 4 that pretreatment with the anti-LDLR antibody prevented senescence induction in HUVECs.

### 3.6. Native LDL-Induced Senescent Cells Were Arrested Permanently at G1 Phase

Although G1-arrested young cells can be reactivated to proliferating cells by treatment with serum or other mitogenic agents, senescent cells theoretically cannot overcome the G1 checkpoint. In line with this, we tried to determine whether the nLDL-induced G1 phase arrest of HUVECs was a temporary or a permanent phenomenon. As a matter of convenience, we wanted to show the irreversibility of cellular senescence (i.e., G1 arrest) induced by the lowest concentration of nLDL. If cellular senescence induced by the lowest concentration of nLDL (2 *μ*g/mL) was not reversed, cellular senescence by higher concentrations of nLDL would be irreversible. After inducing cellular senescence by nLDL treatment (2 *μ*g protein/mL) with subculture for up to 9 days (first cycle of subculture), the cells were washed with EGM-2 medium twice and subcultured again in the same medium without nLDL for up to 6 days (second cycle of subculture). We thought that subculture for 6 days would be enough to show the cell proliferation trend at in vitro culture condition. Flow cytometric cell cycle analysis with PI was carried out subsequently.

The first cycle of subculture with nLDL treatment increased the distribution of G1 phase cells, as compared to the nLDL-untreated cells (Figure 3). After washing out nLDL, the second cycle of subculture for up to 6 days failed to return the cells to their original proliferative state (*p* > 0.05; Table 1). The G1-arrest of HUVECs induced by the lowest concentration of nLDL (2 *μ*g protein/mL) was not reversed by washing out nLDL from the cells. This result indicated that nLDL-induced senescence of cultured endothelial cells was an irreversible change.

### 3.7. Intracellular ROS Generation Was Implicated in NLDL-Induced Cellular Senescence

In this experiment, we determined the role of ROS in the nLDL-induced senescence of HUVECs. The cells were treated with nLDL (0, 2, 5, and 10 *μ*g protein/mL) concomitantly with media exchange in a subculture system for up to 9 days. ROS generation in the nLDL-treated cells was measured by spectrofluorometry (Figure 6(a)) and fluorescence microscopy (Figure 6(b)) with DCF-DA as a chemical probe. The intracellular ROS generation was significantly increased in the nLDL-treated cells dose- and time-dependently (*p* < 0.01). Cells treated with nLDL for 3 days in a subculture system showed no significant ROS generation by independent *t*-test. Nevertheless, cells treated with nLDL for 6 or 9 days showed a significant increase in ROS generation (*p* < 0.05 or 0.01) for all concentrations tested.

## 4. Discussion

In this study, we used both a subculture system and a continuous culture system to eliminate the variables that could occur from each culture system for up to 9 days of culture. Long-term treatment with low concentrations of nLDL (2~10 *μ*g protein/mL) inhibited the proliferation of HUVECs in both culture systems. The inhibition of cell proliferation was shown after 2 days of nLDL treatment, suggesting that as the concentrations of nLDL were very low, the effect required amplification within the cells. The result that the endothelial cell's senescence induced by nLDL treatment preceded their decreased proliferation suggested that the cellular senescence might be responsible for the decreased cellular proliferation. We first showed that nLDL could induce premature senescence of cultured cells.

The cellular distribution to G1 phase was increased by nLDL treatment. The nLDL-induced senescent cells were arrested at the G1 phase of cell cycle. Moreover, the senescent cells did not escape from G1-arrest even on consecutive subculture for up to 6 days after the removal of nLDL. These results suggest that the nLDL-induced G1-arrest of HUVECs is permanent and irreversible.

The oxidative status of nLDL itself is very important in this study, since oxLDL might induce premature senescence of cultured cells. The oxidative status of nLDL isolated from the serum of healthy men has been found to be variable. Colas et al. [42] reported that the degree of lipid peroxidation of LDL was about 245 fmol malondialdehyde (MDA)/*μ*g LDL protein (49.5 pmol MDA/mg cholesterol). However, Han and Pak [43] reported a much lower degree of lipid peroxidation (0.8 fmol MDA/*μ*g LDL protein). The lipid peroxidation level of nLDL used in this study was 8.4 fmol MDA/*μ*g LDL protein. The oxidative status of the nLDL used in this study was about 30 times lower than the value reported by Colas et al. [42] but about 10 times higher than the value reported by Han and Pak [43]. Thus, the nLDL used in this study is within the normal range of oxidative status for healthy men.

Moreover, because nLDL could be oxidized to oxLDL during in vitro culture, we needed to ensure that the senescence was induced not by oxLDL but by nLDL itself. For this purpose, we blocked the receptor for nLDL (LDLR) with the anti-LDLR antibody before nLDL treatment as described by Allen et al. [44], on the basis that oxLDL does not bind to LDLR [45]. Anti-LDLR antibody pretreatment suppressed the nLDL-induced HUVECs senescence. This result suggested that nLDL itself may be endocytosed into the cultured endothelial cells to induce premature senescence of the cells. There is another subfraction of LDL, minimally modified LDL (mmLDL), that is sufficiently modified to be chemically distinguished from nLDL. Despite its modification, mmLDL retains the ability to bind to LDLR [46]. Thus far, this unusual character of mmLDL makes it hard to differentiate the effect of nLDL from that of mmLDL. Further studies are required to solve this problem.

Next, we tried to identify the signal transduction pathway involved in the nLDL-induced endothelial senescence. Growth arrest of senescent cells is maintained by the p53 and/or p16-pRb signal transduction pathways [47–49]. Hence, we conducted Western blot analysis of some of the cell cycle-regulating proteins that are related to these signal transduction pathways and the G1 arrest of senescent cells. The level of p53 was increased. In addition, the level of the dephosphorylated form of pRb was increased, but that of phosphorylated pRb at S807 and S811 [P-pRb (pS807/pS811)] was decreased, indicating that suppression of the phosphorylation of the pRb protein is the cause of senescence induction in the cells. The level of CDK inhibitors such as p21 and p16 was also increased, but the level of cyclin/CDK complex proteins such as Cdk2, Cdk4, Cyclin E2, and Cyclin D1 was decreased. These changes in the level of the cell cycle-regulating proteins in the nLDL-treated cells suggested that nLDL-induced G1 arrest of HUVECs could be due to the inhibition of pRb phosphorylation through both the p53 and p16-pRb signal transduction pathways.

Pretreatment with the anti-LDLR antibody restored in varying degrees the changes in the level of the cell cycle-regulating proteins induced by nLDL treatment. This result corroborated the results of Figure 4 that pretreatment with anti-LDLR antibody prevented nLDL-induced senescence induction in HUVECs.

We also showed that long-term treatment with low concentrations of nLDL could stimulate the generation of ROS in HUVECs. This result suggested that ROS might be implicated in the premature senescence of cultured endothelial cells by nLDL treatment.

The senescence-inducing pathways could be initiated by diverse stressful conditions such as telomere shortening, DNA damage, oncogene activation, lack of nutrients or growth factors, and oxidative stress [47, 49]. Native LDL reportedly can generate superoxide radical (O_2_^−∙^) instead of NO in endothelial cells or tissues, including HUVECs, by uncoupling of eNOS [50–53]. Relatively high concentrations of nLDL (2.4 mg cholesterol/mL) were used in these studies, to show the stimulating effect of nLDL on the generation of ROS in HUVECs. Here, we demonstrated that the premature senescence of the endothelial cells could be induced by long-term treatment, at very low concentrations of nLDL (4.4~22.2 *μ*g cholesterol/mL).

Collectively, our results suggested that long-term treatment with low concentrations of nLDL could induce premature senescence of cultured endothelial cells. Native LDL is endocytosed into the cells through the LDLR and probably generates ROS to induce cellular senescence via both p53 and p16-pRb signal transduction pathways.

The findings from this study are not applicable to in vivo human pathophysiology, since in terms of antioxidant capacity in vitro culture conditions are quite different from in vivo cellular environment. Nevertheless, the nLDL-induced senescence of cultured HUVECs could be used as a model system for the study of premature cellular senescence in in vitro aging conditions. The human body, including the circulatory system, has well-developed homeostatic defense systems that can protect from oxidative stress. Nevertheless, when the homeostatic balance between prooxidant and antioxidant systems in the plasma and endothelial cells is disturbed for a relatively long period, nLDL as well as modified LDL, such as oxLDL, could induce the premature senescence of vascular endothelial cells.

In summary, this is the first report describing nLDL-induced senescence of vascular endothelial cells, at least, partly via oxidative stress under in vitro culture conditions. The nLDL-induced senescence of vascular endothelial cells could be used as a model system for in vitro aging studies. Further studies are required to apply this finding to in vivo human pathophysiology.

## Figures and Tables

**Figure 1:**
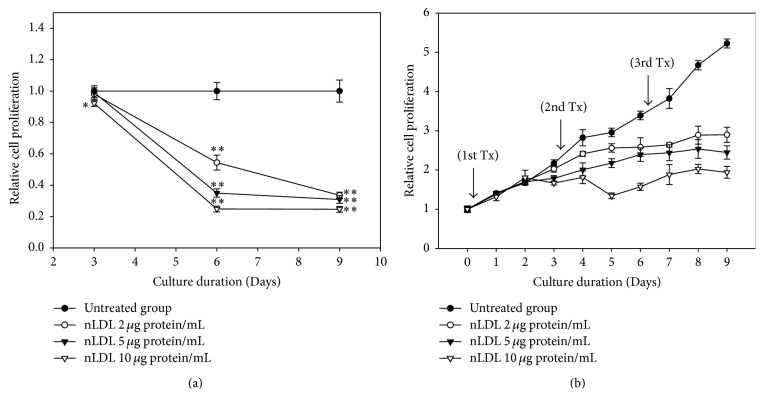
Effect of long-term treatment with nLDL on the proliferation of HUVECs. Young HUVECs (PDL, 12~15) were subcultured at every third day of each subculture with media exchange (a) and cultured continuously in the same culture dish with media exchange (b), for up to 9 days. The cells were treated with various concentrations of nLDL (0, 2, 5, and 10 *μ*g protein/mL) concomitantly with media exchange every 3 days at both culture systems (1st, 2nd, and 3rd Tx). Cellular proliferation of the cells was analyzed by tetrazolium salt method. The degree of cellular proliferation was expressed as the relative ratio of cell number. The dose- and time-dependent differences in cellular proliferation between groups were analyzed statistically by repeated measures ANOVA assay (*p* < 0.01). Each nLDL-treated group was also compared with the respective nLDL-untreated group by independent *t*-test. ^*∗*^*p* < 0.05; ^*∗∗*^*p* < 0.01. Each result represents the mean ± SD (*n* = 6).

**Figure 2:**
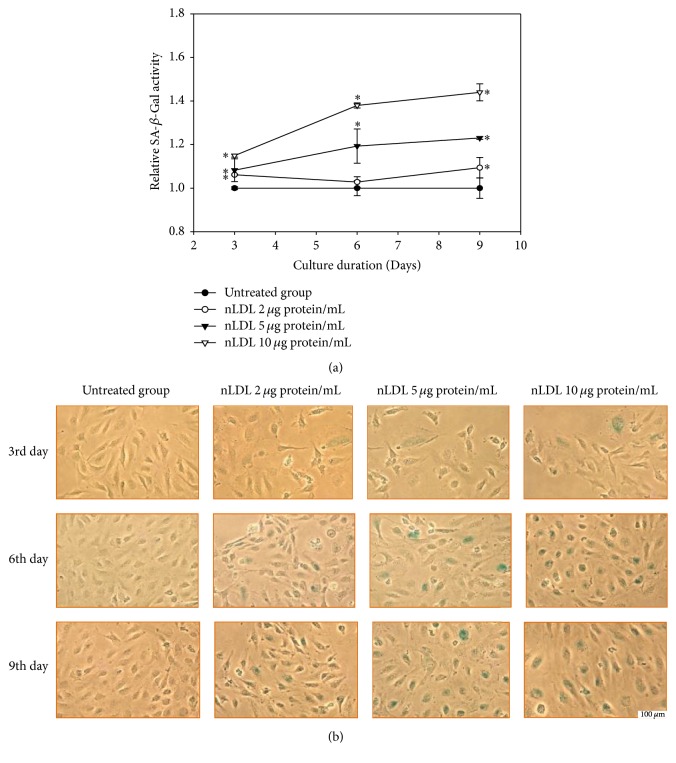
Senescence induction in HUVECs by long-term treatment with nLDL (0, 2, 5, and 10 *μ*g protein/mL), for up to 9 days. (a) Quantitative assay of SA-*β*-Gal activity after nLDL treatment; (b), SA-*β*-Gal activity staining after nLDL treatment. HUVECs were treated with nLDL at the start of each subculture and assayed for SA-*β*-Gal activity at the end of each subculture. The SA-*β*-Gal activity was expressed as the generation rate of 4-methylumbelliferone (MU)/*μ*g protein against that of the respective nLDL-untreated group. The dose- and time-dependent differences in SA-*β*-Gal activity between groups (a) were an alyzed statistically by repeated measures ANOVA assay (*p* < 0.01). Each nLDL-treated group was also compared with the respective nLDL-untreated group by independent *t*-test (^*∗*^*p* < 0.01). Each result represents the mean ± SD (*n* = 3).

**Figure 3:**
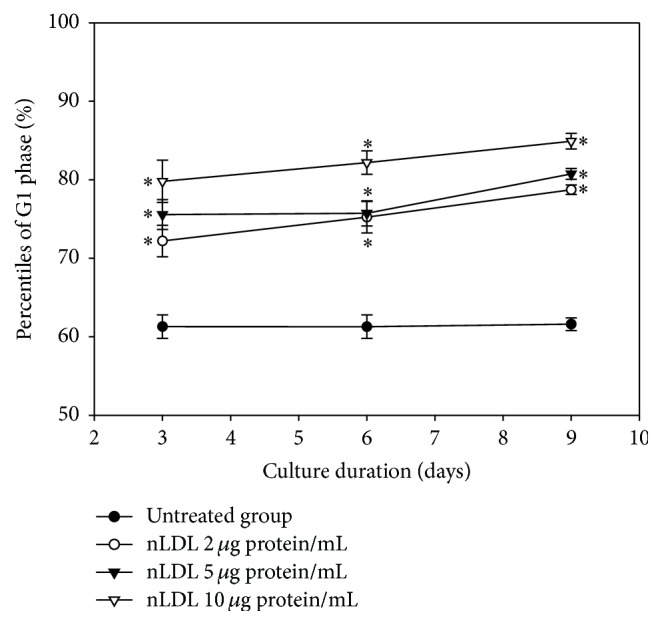
G1 arrest induction in HUVECs by long-term treatment with nLDL. Cell cycle was assayed by flow cytometry in the nLDL-treated cells, for up to 9 days, at the end of each subculture. The distribution percentiles of G1 phase cells after nLDL treatment (0, 2, 5, and 10 *μ*g protein/mL) were shown as a line graph. The dose- and time-dependent differences in G1 phase distribution between groups were analyzed statistically by repeated measures ANOVA assay (*p* < 0.01). Each nLDL-treated group was also compared with the respective nLDL-untreated group by independent *t*-test (^*∗*^*p* < 0.01). Each result represents the mean ± SD (*n* = 6).

**Figure 4:**
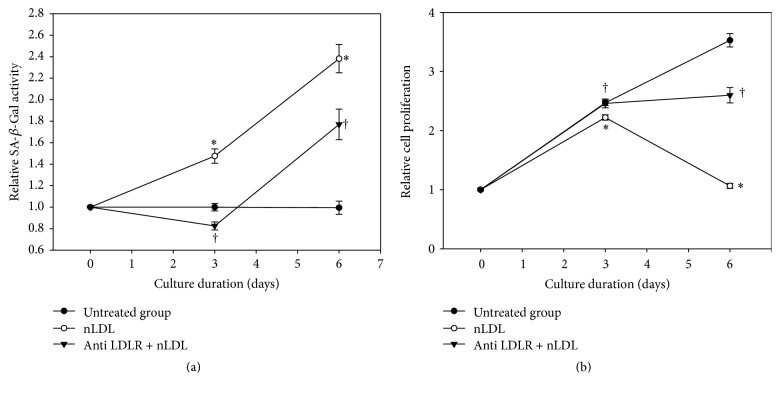
Effect of LDL receptor (LDLR) blocking with antibody on the nLDL-induction of senescence in HUVECs. The cells were pretreated with anti-LDLR antibody (20 *μ*g protein/mL) before nLDL (10 *μ*g protein/mL) treatment. The cells were cultured for up to 6 days. Senescence induction was carried out by SA-*β*-Gal activity assay in subcultured cells (a) and cellular proliferation by tetrazolium salt staining in continuously cultured cells (b), every 3 days. The time-dependent difference in SA-*β*-Gal activity or cellular proliferation between treatment groups was analyzed statistically by repeated measures ANOVA assay (*p* < 0.01). And also, each nLDL-treated group was compared with the respective nLDL-untreated group (^*∗*^*p* < 0.01) and each anti-LDLR antibody plus nLDL-treated group was compared with the respective nLDL-treated group (^†^*p* < 0.01) by independent *t*-test. Each result represents the mean ± SD (*n* = 6).

**Figure 5:**
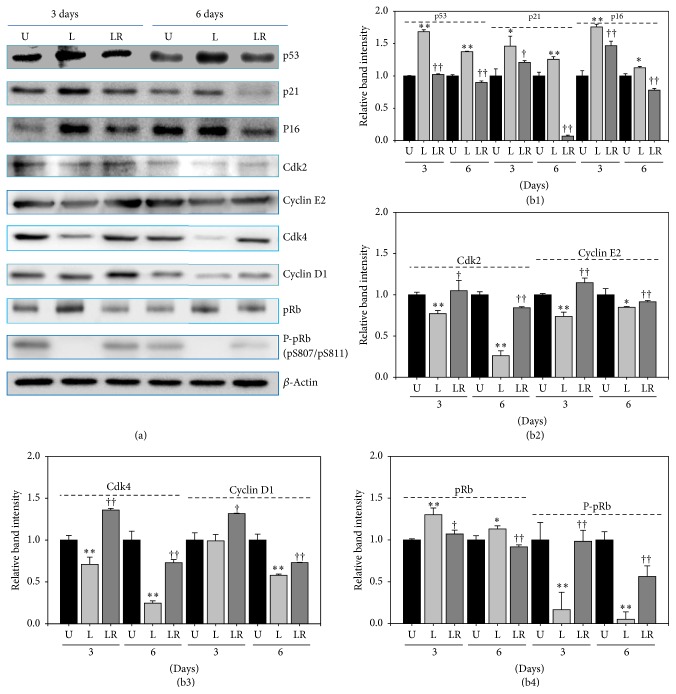
Changes in the content of some cell cycle-regulating proteins by long-term treatment with nLDL (10 *μ*g protein/mL) in HUVECs and their restoration by pretreatment with anti-LDLR (20 *μ*g protein/mL). The cells were untreated with nLDL (U), treated with nLDL (L), or treated with both anti-LDLR and nLDL (LR) at each subculture, for up to 6 days. At the end of each subculture, the content of some cell cycle-regulating proteins was assayed by Western blot analysis. (a) Western blot images; (b) quantification of Western blot bands. (b1) The contents of p53, p21, and p16 proteins were increased by nLDL treatment, with these effects compromised by the anti-LDLR pretreatment. (b2) The nLDL-induced decrease in Cdk2 and Cyclin E2 was compromised by the anti-LDLR pretreatment. (b3) The nLDL-induced decrease in Cdk4 and Cyclin D1 was compromised by anti-LDLR pretreatment. (b4) The content of pRb was increased and that of P-pRb (phosphorylated pRb) was decreased, with these effects compromised by the anti-LDLR pretreatment. Group L was compared with the nLDL-untreated group (U) by independent *t*-test (^*∗*^*p* < 0.05 and ^*∗∗*^*p* < 0.01). Group LR was compared with group L by independent *t*-test (^†^*p* < 0.05 and ^††^*p* < 0.01).

**Figure 6:**
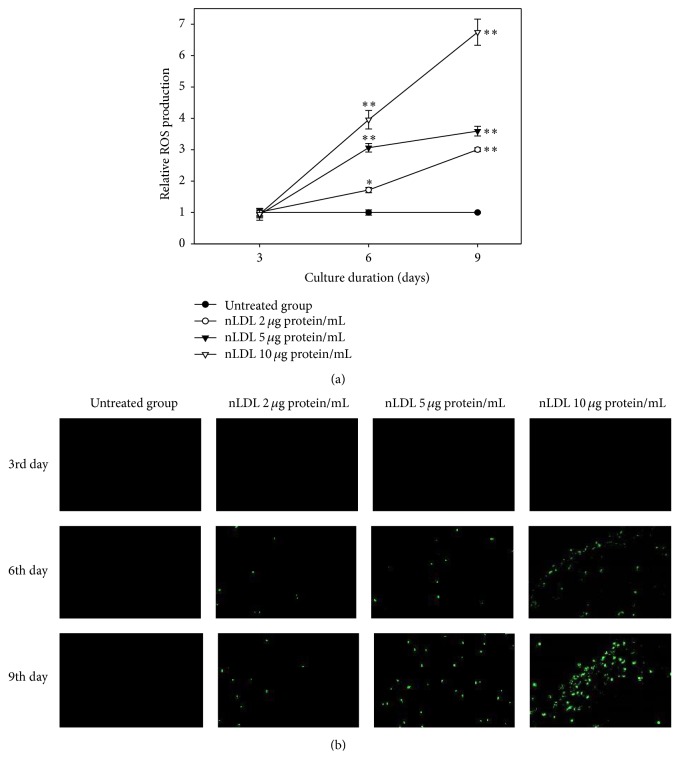
The generation of reactive oxygen species (ROS) with nLDL in HUVECs. The cells were subcultured and treated with nLDL (0, 2, 5, and 10 *μ*g protein/mL) every 3 days of each subculture, for up to 9 days. ROS generation in nLDL-treated cells were analyzed by spectrofluorometry (a) and fluorescence microscopy (b) assays with DCF-DA at the end of each subculture. ROS generation was expressed as the relative fluorescence intensity/*μ*g of protein of the nLDL-treated group, as compared to the untreated group. The dose- and time-dependent differences in ROS generation (a) between groups were analyzed statistically by repeated measures ANOVA assay (*p* < 0.01). Each nLDL-treated group was also compared with the respective nLDL-untreated group by independent *t*-test (^*∗*^*p* < 0.05 and ^*∗∗*^*p* < 0.01). Each result represents the mean ± SD (*n* = 3-4).

**Table 1:** Native LDL-induced G1 arrest of HUVECs was not reversed by nLDL removal. The cells were treated with nLDL (2 *μ*g protein/mL) for 3, 6, and 9 days (first cycle of subculture) and the G1-arrested cells were subcultured again for 3 and 6 days without nLDL (second cycle of subculture). Cell cycle was analyzed by flow cytometry at the end of second subculture. The 3 and 6 days groups of the second cycle of subculture were compared with the 0 day group of the second cycle of subculture by independent *t*-test (*p* > 0.05). Each result represents the mean ± SD (*n* = 3-4).

First cycle of subculture of nLDL pretreatment (2 *μ*g protein/mL)	Second cycle of subculture after nLDL removal	G1 phase	*p* value
3 days	0 days	76.2 ± 0.7	*p* > 0.05
	3 days	76.3 ± 1.7	
	6 days	77.8 ± 4.5	

6 days	0 days	77.1 ± 0.7	*p* > 0.05
	3 days	77.7 ± 0.9	
	6 days	79.3 ± 1.9	

9 days	0 days	80.7 ± 0.7	*p* > 0.05
	3 days	82.5 ± 0.9	
	6 days	84.8 ± 2.6	
